# Преодоление резистентности пролактином к медикаментозной терапии: от перспектив к реальной клинической практике

**DOI:** 10.14341/probl13351

**Published:** 2024-01-24

**Authors:** А. С. Шутова, Е. А. Пигарова, Л. И. Лепешкина, В. А. Иоутси, М. Ю. Дроков, С. Ю. Воротникова, Л. И. Астафьева, Л. К. Дзеранова

**Affiliations:** Национальный медицинский исследовательский центр эндокринологии; Национальный медицинский исследовательский центр эндокринологии; Российский университет дружбы народов имени Патриса Лумумбы; Национальный медицинский исследовательский центр эндокринологии; Национальный медицинский исследовательский центр гематологии; Национальный медицинский исследовательский центр эндокринологии; Национальный медицинский исследовательский центр нейрохирургии им. акад. Н.Н. Бурденко; Национальный медицинский исследовательский центр эндокринологии

**Keywords:** аденома гипофиза, пролактинома, резистентность, хромато-масс-спектрометрия, персонализация лечения

## Abstract

Основной метод лечения пролактин-секретирующих аденом гипофиза — терапия агонистами дофамина, в большинстве случаев позволяющая нормализовать уровень пролактина и уменьшить размеры аденомы. Однако значительная часть пациентов — около 20% неудовлетворительно реагируют даже на высокие дозы агонистов дофамина, что обусловлено резистентностью к терапии. Одной из причин развития резистентности к стандартному лечению является наличие ряда фармакодинамических характеристик. Клинические проявления персистирующей гиперпролактинемии обусловлены двумя патологическими факторами: гормональной гиперпродукцией и масс-эффектом аденомы гипофиза.

Предотвращение необратимых изменений возможно только при своевременном выявлении резистентности и определении оптимального персонализированного алгоритма лечения. Нами описан клинический случай опухолевой гиперпролактинемии, резистентной к терапии агонистами дофамина. Назначение комбинированного лечения тамоксифеном и агонистами дофамина позволило достичь улучшения состояния здоровья пациентки, нормопролактинемии и уменьшения размеров аденомы гипофиза. Гиперпролактинемия, обусловленная пролактин-секретирующей аденомой гипофиза, и ассоциированные с ней патологические проявления представляют собой значимую проблему, неблагоприятно влияющую на качество жизни отдельного пациента и демографическую ситуацию в целом. Это подчеркивает важность изучения причин и выявления предикторов резистентности. Результаты проводимого исследования, иллюстрированные клиническим примером, представлены в настоящей работе.

## Abstract (en)

The main treatment option of prolactin-secreting pituitary adenomas is dopamine agonist therapy, which demonstrates prolactin level normalizing and reducing the size of an adenoma in the majority of cases. However, significant amount of patients — about 20% — poorly responds even to high doses of dopamine agonists that is explained by the resistance to therapy. The occurrence of pharmacodynamic characteristics is one of the causes responsible for the development of resistance to typical therapy. Clinical manifestations of persistent hyperprolactinemia are due to following pathological factors: hormonal hypersecretion and the mass-effect of pituitary adenoma. Prevention of irreversible changes is possible only with timely detection of resistance and determination of the optimal personalized treatment algorithm.We report a clinical case of dopamine-agonist resistant microprolactinoma. Patient’s health stabilisation, normal level of prolactin and reduction in size of adenoma were achieved due to administration of combined treatment with tamoxifen and dopamine agonists. Hyperprolactinaemia occurring because of prolactin-secreting pituitary adenoma and associated adverse effects are significant problem, decreasing quality of life and demographics in general. This underlines the importance of figuring out causes and identifying predictors of the therapy resistance.The results of the study, illustrated by a clinical example, are presented in the present paper.

## ВВЕДЕНИЕ

Этиологическая характеристика гиперпролактинемии многообразна, при этом значительная часть приходится на опухолевую, обусловленную пролактиномами [1]. Пролактин-секретирующие аденомы являются самыми частыми среди всех нейроэндокринных опухолей гипофиза, на их долю приходится примерно 40% [2][3]. Распространенность пролактином у мужчин составляет 10 случаев, а у женщин они встречаются значимо чаще — 50 случаев на 100 000 населения [1].

Клинические проявления опухолевой гиперпролактинемии ассоциированы с гормональной гиперпродукцией и масс-эффектом аденомы гипофиза. Каждый из этих факторов вносит вклад в возникновение необратимых, зачастую жизнеугрожающих изменений, в связи с чем важна разработка алгоритма своевременной диагностики и персонализированного лечения. Препаратами выбора при лечении гиперпролактинемии любого генеза, в том числе опухолевого, являются агонисты дофаминовых рецепторов, наиболее эффективный и безопасный из которых — каберголин [4–6]. Он стимулирует дофаминовые рецепторы 2 типа (D2-рецепторы), мимикрируя физиологическое действие дофамина по подавлению секреции пролактина в лактотрофах. Каберголин обладает синергическим двунаправленным эффектом — антисекреторным и антипролиферативным. Результатом является снижение уровня пролактина в сыворотке крови, восстановление репродуктивной функции и уменьшение размеров аденомы гипофиза.

Однако, согласно клиническим рекомендациям, в 15–20% случаев не достигается должного эффекта, несмотря на прогредиентное повышение дозы каберголина (4,5 мг в неделю — максимально рекомендуемая доза в инструкции) [1]. Резистентность к лечению определяется как отсутствие нормализации уровня пролактина и/или уменьшения размеров пролактиномы на 50% и более после полугодового лечения каберголином в дозе не менее 3 мг в неделю [7][8]. Предполагаемыми причинами резистентности могут быть нарушения абсорбции, распределения и метаболизма каберголина (в том числе генетически опосредованные), а также рецепторные и пострецепторные изменения в самой опухолевой ткани, своевременное выявление которых поможет выбрать оптимальное лечение. Преодоление резистентности позволит предотвратить развитие патологических проявлений, таких как бесплодие, потеря зрения вследствие повреждения зрительных нервов, гипопитуитаризм.

Вероятность развития осложнений гиперпролактинемии, увеличивающаяся пропорционально периоду неэффективного лечения, обусловливает целесообразность неординарного подхода к тактике лечения резистентных пациентов, в том числе с назначением препаратов off-label. Для иллюстрации представляем данное клиническое наблюдение.

## ОПИСАНИЕ СЛУЧАЯ

Пациентка Н., 39 лет, впервые обратилась в ГНЦ ФГБУ «НМИЦ эндокринологии» Минздрава России с жалобами на выраженную непрекращающуюся боль и дискомфорт в области молочных желез, повышение утомляемости на фоне привычной физической нагрузки, ежедневные эпизоды головной боли, выпадение волос и отсутствие либидо. Менструальный цикл отсутствовал вследствие ранее проведенной гистерэктомии с резекцией левого яичника по поводу множественной миомы матки и кисты яичника.

Впервые ухудшение самочувствия отмечено двумя годами ранее, проявлялось общей слабостью, апатией, увеличением выраженности ранее отмеченных симптомов, а также увеличением массы тела на 8 кг за 6 мес, несмотря на снижение калорийности суточного рациона питания. Пациентка консультирована эндокринологом, установлено повышение пролактина в сыворотке крови до 50,5 нг/мл (при референсных значениях до 26,7 нг/мл), инициирована терапия каберголином 0,5 мг по ½ таблетке дважды в неделю. По данным проведенной МРТ головного мозга с контрастированием выявлена микроаденома гипофиза размерами 2,5х3х1,5 мм.

Несмотря на регулярный прием препарата, достичь снижения уровня пролактина в сыворотке крови не удавалось. Постепенно доза каберголина увеличена до 4,5 мг в неделю, однако положительная динамика клинико-лабораторных показателей и состояния здоровья пациентки не была достигнута.

Учитывая отсутствие эффекта от терапии агонистами дофамина, пациентка госпитализирована в отделение нейроэндокринологии ГНЦ ФГБУ «НМИЦ эндокринологии» Минздрава России, при поступлении подтверждена гиперпролактинемия: пролактин — 1900 мЕд/л (биоактивный пролактин — 1780 мЕд/л), выявлена двусторонняя галакторея 2 cтепени, болезненность молочных желез при пальпации, избыточная масса тела (ИМТ 28,7 кг/м²). Выполненная МРТ головного мозга с контрастированием демонстрировала отрицательную динамику по сравнению с предыдущим исследованием (11 мес назад) в виде увеличения размеров аденомы гипофиза до 3,5х3х2 мм.

Установлен диагноз: Пролактин-секретирующая эндоселлярная микроаденома гипофиза. Опухолевая гиперпролактинемия, резистентность к лечению агонистами дофамина.

В связи с предшествующим назначением максимальной дозы каберголина, на основании проведенного консилиума принято решение о подключении к терапии бромокриптина в дозе 1,25 мг 2 раза в день с постепенным повышением дозы до 2,5 мг дважды в сутки. На фоне приема сочетанной терапии в течение 12 мес отмечено снижение уровня пролактина в сыворотке крови (пролактин 740 мЕд/л), однако референсные значения достигнуты не были, самочувствие со слов пациентки — с улучшением: отмечено повышение работоспособности и толерантности к физическим и эмоциональным нагрузкам, уменьшение выраженности дискомфорта в области молочных желез.

Учитывая проведенную ранее гистерэктомию и ожидаемый потенцирующий эффект снижения эстрогенового влияния как на уровне гипофиза, так и молочных желез, к сочетанной терапии каберголином и бромокриптином добавлен селективный модулятор эстрогеновых рецепторов тамоксифен. При динамическом обследовании через 6 мес комбинированной терапии отмечена нормализация уровня пролактина в сыворотке крови, отсутствие галактореи, нагрубания молочных желез.

Принимая во внимание выраженную положительную динамику комбинированной терапии, при очередной госпитализации в ГНЦ ФГБУ «НМИЦ эндокринологии» Минздрава России предпринята попытка уменьшения дозы агонистов дофамина. Постепенно отменен каберголин, сохранен прием бромокриптина 1,25 мг 2 раза в сутки и тамоксифена 20 мг ежедневно. На фоне проводимой терапии отмечалась стабильная нормопролактинемия (при двукратном исследовании через 6 мес — 268 и 301 мЕд/л соответственно), отсутствовали галакторея и чувство нагрубания молочных желез, снизилась масса тела на 10 кг, прекратилась выраженная головная боль. Прием бромокриптина совместно с тамоксифеном был продолжен, так как попытка его отмены сопровождалась повышением уровня пролактина. Графически указанные изменения отражены на рисунке 2.

Известно, что одной из причин резистентности к терапии агонистами дофамина является наличие фармакодинамических особенностей. С целью оценки метаболомных характеристик пациентке проведена проба с каберголином, результаты которой подтвердили наличие предполагаемых метаболомных дефектов — концентрация каберголина в сыворотке крови достигала закономерного пика, однако ожидаемое плато концентрации препарата, опосредованное длительным периодом его полувыведения, впоследствии не наблюдалось (табл. 1, рис. 1).

**Table:** Таблица 1. Метаболомные характеристики пациентки Н.

Параметр	Концентрация каберголина, интенсивность, приблизительные единицы
Процент прироста концентрации каберголина, %	152
Максимальная концентрация каберголина в сыворотке крови, пг/мл	64,4
Минимальная концентрация каберголина, пг/мл	19,3
Время пика каберголина, мин	120
Средняя концентрация каберголина, пг/мл	39
Продолжительность плато концентрации каберголина, мин	60
Время начала снижения концентрации каберголина, мин	120
Процент убыли концентрации каберголина, %	-54

**Figure:**
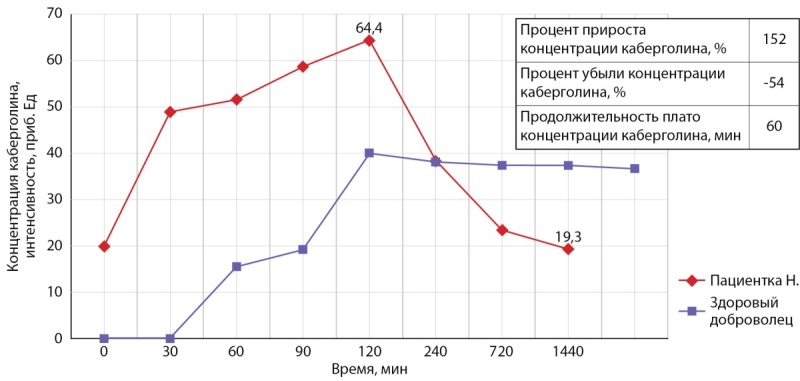
Рисунок 1. Метаболомные характеристики пациентки Н.

**Figure:**
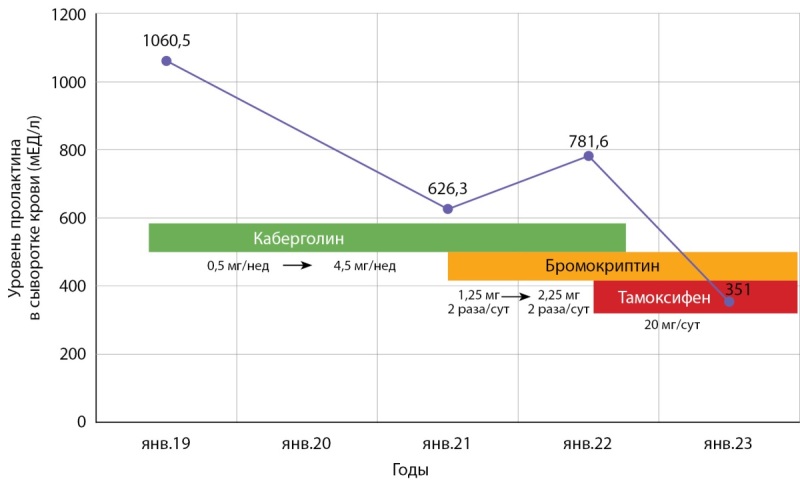
Рисунок 2. Динамика изменения уровня пролактина в сыворотке крови пациентки Н. на фоне комбинированной терапии.

При МРТ головного мозга, проведенной в динамике на фоне снижения дозы каберголина, визуализировалась микроаденома гипофиза размерами 2,5×2×2 мм: таким образом, ранее наблюдавшаяся отрицательная динамика в виде увеличения размеров аденомы отсутствовала. В течение периода терапии тамоксифеном пациентке регулярно проводилось цитологическое исследование мазков из шейки матки, по результатам которого признаков патологического процесса не выявлено.

## ОБСУЖДЕНИЕ

Учитывая высокую встречаемость пролактин-секретирующих аденом, значительный вклад данного заболевания в уменьшение репродуктивного потенциала популяции и снижение качества жизни, решение проблемы гиперпролактинемии является важным аспектом общественного здоровья и позитивного тренда демографической ситуации [9][10]. В настоящее время возможности применения альтернативных методов лечения у пациентов, резистентных к агонистам дофамина, ограничены: лучевая терапия малоэффективна [11][12], радикальная операция в большинстве случаев невозможна в связи с тенденцией пролактин-секретирующих аденом гипофиза к параселлярному распространению и экспансивному росту [6]. Таким образом, наиболее перспективным направлением в ведении резистентных пациентов является применение медикаментозной терапии, в том числе — сочетанной и комбинированной.

Учитывая вероятное наличие дефектов метаболизма агонистов дофамина, пациентке проведена фармакокинетическая проба, результаты которой подтвердили патогенетическую обоснованность назначенной сочетанной терапии каберголином и бромокриптином. Выявленный патологический метаболический паттерн пациентки, соответствующий фенотипу «поднимает и роняет», диктует необходимость специфической коррекции терапии. Чередование виража концентрации препарата с последующим снижением практически до базового уровня и отсутствие поддержания постоянной концентрации препарата в сыворотке крови обусловливали невозможность реализации терапевтического эффекта препарата. Комбинация каберголина и короткодействующего агониста дофамина — бромокриптина позволяет поддерживать концентрацию агониста дофамина в сыворотке крови пациентки на относительно постоянном уровне. Назначенная сочетанная терапия сопровождалась предсказуемым положительным эффектом в виде снижения уровня пролактина в сыворотке крови, отсутствия галактореи и болезненности молочных желез, урежения частоты эпизодов головной боли. Учитывая выраженные позитивные клинические и лабораторные изменения, перспективы достижения нормопролактинемии и полного исчезновения негативных проявлений заболевания, для полноценной реализации потенциала консервативного метода лечения принято решение об эскалации терапии в виде присоединения к терапии селективного модулятора эстрогеновых рецепторов — тамоксифена [7][13–17].

Известно, что прием тамоксифена ассоциирован с повышенным риском развития как доброкачественных, так и злокачественных новообразований эндометрия. Проведенная гистерэктомия исключила необходимость в настороженности в отношении увеличения пролиферативной активности эндометрия, что стало ключевым фактором в выборе дополнительного медикаментозного агента [7][18].

В литературе описаны случаи комбинированного назначения агонистов дофамина и селективного модулятора эстрогеновых рецепторов тамоксифена. Среди таковых следует отметить уникальный клинический случай Z.K. Christian и соавт., описывающих пациентку с агрессивной пролактин-секретирующей макроаденомой гипофиза и персистирующей гиперпролактинемией, несмотря на проведенную двукратную транссфеноидальную аденомэктомию, лобно-височную краниотомию, лучевую терапию на аппарате «гамма-нож» и прием каберголина в дозе 4,5 мг в неделю [13]. Клиническая картина сопровождалась негативной неврологической симптоматикой — изнуряющей головной болью, сужением полей зрения, характерными проявлениями гиперпролактинемии. Спустя 6 мес после назначения комбинации каберголина и тамоксифена уровень пролактина в сыворотке крови пациентки снизился до 3 нг/мл, по данным МРТ головного мозга достигнута положительная динамика в виде уменьшения размеров аденомы [13]. Продолжительность последующего наблюдения пациентки составила 14 лет, на протяжении которых отмечалось сохранение нормопролактинемии, отсутствие признаков аденомы гипофиза согласно МРТ [13]. Важное значение имеет проспективное одноцентровое исследование Н.С. Федоровой и соавт., посвященное изучению возможностей комбинированной терапии в лечении пациентов с пролактиномами, резистентными к терапии [7]. В работу включены женщины в возрасте 23–38 лет с пролактин-секретирующими аденомами гипофиза, не достигшие положительного эффекта на фоне длительного приема каберголина в максимальной и субмаксимальной дозе [7]. В дополнение к каберголину пациенткам назначался тамоксифен 20 мг/сут с повышением дозы до 40 мг/сут при отсутствии нормализации пролактина в сыворотке крови через 1 мес. Спустя 3 мес комбинированной терапии у 100% пациенток наблюдалось статистически значимое снижение уровня пролактина на 22–66%. Уменьшение дозы тамоксифена в связи с увеличившейся пролиферативной активностью эндометрия потребовалось только одной пациентке [7].

Также следует упомянуть публикацию J. Lopez и соавт., демонстрирующую случай опухолевой гиперпролактинемии у резистентной к агонистам дофамина пациентки, основными жалобами которой были аменорея и бесплодие, на фоне исключения гинекологических причин и относительного здоровья супруга [15]. Пациентке проводилась длительная, но безуспешная терапия различными агонистами дофамина (по отдельности и в сочетании): бромокриптином, каберголином и хинаголидом. Достичь восстановления менструального цикла и возникновения овуляции позволило назначение комбинации тамоксифена 20 мг/сут и бромокриптина 5 мг/сут с последующим повышением до 7,5 мг/сут. На фоне терапии отмечено снижение уровня пролактина в сыворотке крови, и, несмотря на отсутствие достижения референсных значений показателя, у пациентки восстановился регулярный менструальный цикл и подтверждено наличие овуляции [15].

Показательно, что в каждом из описанных клинических примеров на фоне комбинированной медикаментозной терапии достигнут положительный эффект.

В рассматриваемом нами случае на фоне комбинированной терапии предпринималась попытка отмены агонистов дофамина, однако с неудовлетворительным эффектом в виде значительного повышения уровня пролактина, что потребовало возобновления терапии. Тем не менее проведенная коррекция позволила экспериментально определить наименьшую дозу агонистов дофамина, необходимую для поддержания нормопролактинемии. В ходе продолжающейся титрации дозы отмечено, что наибольшую эффективность в отношении купирования боли и нагрубания молочных желез имел бромокриптин, в связи с чем последующее снижение дозы проводилось со стороны менее значимого в этом отношении каберголина. В итоге стала возможной полная отмена каберголина, который ранее пациентка длительное время принимала в максимально допустимой дозе. Примечательно, что на фоне приема минимальной дозы бромокриптина и тамоксифена удалось стабилизировать состояние здоровья с поддержанием нормопролактинемии, отсутствием описанных выше патологических симптомов и прекращением роста опухоли гипофиза.

## ЗАКЛЮЧЕНИЕ

Опухолевая гиперпролактинемия, обусловленная пролактин-секретирующей аденомой гипофиза, и ассоциированные с ней патологические изменения представляют собой значимую медико-социальную проблему, существенно снижают качество жизни отдельного пациента и нарушают демографическую ситуацию в целом. Кроме того, длительный период неэффективного лечения обусловливает негативные топографо-анатомические изменения аденомы, прогрессию неврологических нарушений. Достаточно широкая распространенность резистентности к терапии агонистами дофамина определяет необходимость формирования и верификации подходов к ее преодолению, отсутствующих в настоящее время. Одним из способов решения данной проблемы может являться комбинированное лечение тамоксифеном и агонистами дофамина. Для реализации максимального терапевтического потенциала каберголина целесообразно определение индивидуального фармакокинетического профиля с целью подбора оптимальной дозы и длительности действия препаратов.

## ДОПОЛНИТЕЛЬНАЯ ИНФОРМАЦИЯ

Источники финансирования. Работа подготовлена в рамках гранта Министерства образования и науки Российской Федерации, соглашение 075-15-2022-310 от 20.04.2022.

Конфликт интересов. Авторы декларируют отсутствие явных и потенциальных конфликтов интересов, связанных с содержанием настоящей статьи.

Участие авторов. Шутова А.С. — получение, анализ данных, написание статьи; Пигарова Е.А. — анализ данных, внесение в рукопись правок; Лепешкина Л.И. — анализ данных, написание статьи, Иоутси В.А. — анализ данных, проведение метаболомных исследований; Дроков М.Ю. — внесение в рукопись правок, статистическая обработка; Воротникова С.Ю. — анализ данных, внесение в рукопись правок; Дзеранова Л.К. — анализ данных, внесение в рукопись правок.

Все авторы одобрили финальную версию статьи перед публикацией, выразили согласие нести ответственность за все аспекты работы, подразумевающую надлежащее изучение и решение вопросов, связанных с точностью или добросовестностью любой части работы.

Согласие пациента. Пациентка добровольно подписала информированное согласие на публикацию персональной медицинской информации в обезличенной форме в журнале «Проблемы эндокринологии».
